# Parenting young people with complex regional pain syndrome: an analysis of the process of parental online communication

**DOI:** 10.1097/PR9.0000000000000681

**Published:** 2018-09-11

**Authors:** Kaedi Navarro, Elaine Wainwright, Karen Rodham, Abbie Jordan

**Affiliations:** a Department of Psychology, University of Bath, Bath, United Kingdom; b Department of Psychology, Bath Spa University, Bath, United Kingdom; c Department for Health, University of Bath, Bath, United Kingdom; d Department of Psychology, Staffordshire Centre for Psychological Research, Staffordshire University, Stoke on Trent, United Kingdom; e Department of Psychology and Centre for Pain Research, University of Bath, Bath, United Kingdom

**Keywords:** Parenting, CRPS, Support, Young people, Pediatric pain

## Abstract

**Introduction:**

Parenting a young person with complex regional pain syndrome (CRPS) is associated with high levels of parental distress and numerous emotional, informational, and practical challenges. To meet these challenges, parents seek others undergoing similar experiences, both in face to face and online forums.

**Objectives:**

The objective of this study was to conduct a qualitative analysis of online forum data to explore the process of parental forum communication regarding parenting a young person with CRPS in online spaces.

**Methods:**

A total of 107 forum posts relating to parenting a young person with CRPS were collected from 39 users across 2 public forums. Data were analyzed using thematic analysis.

**Results:**

Findings identified 2 themes: “the informal rules of exchanging and receiving network support” and “parents positioning themselves as experts.” The first theme highlighted the varied nature of support sought and provided by parents in addition to social rules associated with the negotiation of this support. The second theme represented an understanding of how parents presented themselves as experts in their young person's pain, both in relation to fellow parents and health care professionals.

**Conclusion:**

This study provided a novel insight into support and communicational exchanges between parents of young people with CRPS on online public forums. Findings identified the perceived usefulness of online spaces in terms of parents of young people with CRPS seeking and providing support. Further research can helpfully investigate how we might implement online peer mentoring to improve support further for parents.

## 1. Introduction

Complex regional pain syndrome (CRPS) is a pain condition characterized by high levels of pain intensity, automatic, motor and motor impairment.^18^ Complex regional pain syndrome typically affects bodily extremities (arms and legs) and is accompanied by allodynia, hyperaesthesia, and pain-related disability.^20^ Reported pain intensity in CRPS is typically substantially higher than that expected from any causative event (eg, fracture). Although diagnostic criteria for CRPS exist, such as the favored Budapest Criteria,^22^ diagnosis of CRPS is difficult. This is most often delayed because of varied reports of sensory, vasomotor, sudomotor and motor symptom presentation, and the lack of response of CRPS pain to analgesia.^19^ Although more common in adults, CRPS is also diagnosed in young people. Prevalence of CRPS in young people is greatest in females aged 12 years and above,^44^ with high levels of accompanying pain-related disability.^38^ Specifically, young people with CRPS report elevated levels of pain, psychosomatic symptoms, and psychological distress compared to young people with headache or abdominal pain.^26^

Consistent with the broader pediatric pain literature, the impact of CRPS extends beyond the young person, affecting parents and the family.^28,34^ The biopsychosocial framework for pediatric CRPS presents a bidirectional parent–child dyad relationship, with high levels of stress for the parent influencing the psychological, emotional, and social coping strategies of the parent and young person. In turn, this can result in further functional disability for the young person, and consequently, a reduction in effective parental coping strategies, resulting in elevated levels of stress.^13^ The wider pediatric pain literature has established that parents have reported that the chronic uncertainty surrounding the young person's pain condition and parental psychological distress in response to the challenges of obtaining a “correct” diagnosis for their young person.^25,29^ Living with a young person with persistent pain is often challenging, and parents are motivated to seek information and support to enable them to manage these challenges.

Research studies have demonstrated the benefits of seeking support with regard to encouraging good quality parenting and reducing parental impact associated with managing their child's health condition styles.^14^ Such support has historically been sought in face to face support settings, yet with the development of a more digital age, support in terms of managing health-related concerns is increasingly sought online.^21^ Online forums can offer similar therapeutic attributes to face to face support groups,^24^ with research focusing specifically on the role of health-related forums.^8,39^ Forums provide new and easier opportunities for parents and caregivers to obtain information, support, and advice.^3^ In particular, Sullivan^43^ noted the convenience for parents of receiving social support within their own home to share, validate, and normalize their experiences with other parents online, especially if these parents felt isolated as a result of parenting a child with a mental or physical illness.

Research studies have demonstrated the various benefits that individuals gain from seeking online support and how the online forums have shown to be a valuable online peer support environment as opposed to attending a face to face support group.^11,27^ One study involving parents of children with cancer found that parental experience of forum support was mixed.^11^ Parents reported benefit in both receiving varied forms of online forum support (eg, emotional, social, informational, esteem, and tangible assistance) yet were frustrated by their inability to contact forum members offline and the lack of replies on particular threads. With regard to CRPS specifically, only 4 studies exist regarding use of online health forums. However, these relate to adult use of forums rather than parental use.^17,32,40-41^ Little is known concerning how parents of young people with CRPS use forums, and in particular, how support is solicited, offered, and received in these online environments. Consequently, as much is already known about parental impact of parenting a young person with chronic pain,^25^ there is a need to move beyond examining forum content concerning parental impact and experience and to focus on parental communication styles, specifically, how communication is sought, provided, and maintained by parents of young people with CRPS. This study focuses on exploring how parents of children with CRPS communicate online with one another by performing a thematic analysis of interactions between parents' posts on online public forums. With regard to a specific objective, this study will determine and describe the different ways in which parents communicate with one another regarding parenting a young person with CRPS through analysis of data from UK-based parenting and health-related forums.

## 2. Method

### 2.1. Data collection

Online forums were identified using the following key terms: “chronic pain,” “parents,” “experiences,” “pediatric,” (Complex Regional Pain Syndrome) “CRPS,” (Reflex Sympathetic Dystrophy) “RSD,” “children,” and “child” using the Google search engine on April 20, 2017. This identification process has been used in previous online forum research.^4,11,15,23^

The process of selecting forums was conducted by following guidelines from previous relevant studies.^31^ These authors considered an “open message board” to be a public domain and an online forum environment, where people contributing to the forum can be expected to be aware that other contributors or online “lurkers” (a person online observing but not participating in forum discussions) will have access to the messages that have been posted on the forum.^40,41^ In the current study, forums were eligible for inclusion if they had “public access” (visible to anyone with access to the Internet who is not necessarily a member of the online forum). This is consistent with established published ethical guidelines,^16^ which suggest public domain data can be used for research purposes without the need for informed consent from participants. No restriction was placed on host country for the forum.

Forum posts were also only considered if they had a searchable archive that had “inactive” forum threads. A thread is defined as a, “Collection of messages posted as replies to previous messages”^2^ after guidance from Coulson,^10^ forum threads were only selected if the forum website comprised at least 500 members to further ensure the anonymity of posts included in the analyses and subsequent paper (Coulson, 2005). Forums with searchable archives were selected to improve the ease of identification of all relevant threads for analyses.

Three online forums were identified, which adhered to the criteria of (1) forums allowing for access to nonmembers of the websites (2) having a large number of members participating on the forums and (3) the thread topic where parents discussed their young person's CRPS with one another. After asking for permission to use the data from several online forum sites, 2 of the 3 online forum moderators granted permission to use the message posts as data in the study. One forum did not reply to our request. Threads from the eligible forums were included in the analyses if there were a minimum post number of twenty posts per thread to enable the study of interaction between forum members. Consequently, the study comprised analysis of 2 threads discussing individuals' experiences of parenting a child with CRPS, with a total of 107 messages from 2 forums; an online parenting forum and a medical forum focusing on long-term conditions. Both forums were UK-based forums. The number of posts ranged between 2 and 12 posts per participant, with a participant posting a mean of 3.79 posts each on these specific forum threads.

### 2.2. Participants

Twenty participating online members were identified from the first online forum thread and 19 from the second. Concordant with previous online forum studies,^9,10,40^ it was difficult to determine demographic information about the posters because of anonymity issues. A total of 15 forum messages were excluded as the posters described themselves as adults with CRPS. Establishing parents' gender was challenging because of usernames not being gender-specific.

### 2.3. Procedure

Ethical approval was granted by the relevant university ethics committee. In accordance with existing guidelines for conducting online research, permission for use of the data was requested by contacting the site moderators of the online forums.^7^ To protect the confidentiality of the posters and online forums, real forum names, thread-specific details, and usernames are not reported here.^7^ Forum posts occurred over a 2-year period for the parenting forum and over a 1-year period for the health-related forum.

Online usernames and quotations in the study were anonymized to protect the anonymity of posters as direct permission from the posters was not sought to use the data. Usernames were replaced with pseudonyms. Pseudonyms were chosen by the most common female names based on the assumption that most posters were female.^33^ After analyses, after good practice, quotations were also paraphrased and typed into a Google search engine to ensure they could not be retraced to the original source.^4,23^ All changes were recorded, and these included identifying names, medical terms, low-frequency words, adjectives, and nouns. The final version of the anonymized quotations and audit table were reviewed by A.J. and E.W. to ensure that the content and meaning of the quotations remained unchanged despite paraphrasing. In accordance with existing literature, if quotations were still traceable when entered into a Google search engine, they were not included in the study.^31^ This resulted in only one quotation being excluded because it included multiple references to specific treatment centers and a particular health care professional. It was impossible to anonymize without losing meaning.

### 2.4. Data analysis

The message posts were extracted from the online forums using a free online software program Dataminer.^12^ Data (forum posts) including usernames and dates and times of posts were downloaded from Dataminer and converted into a text document. The text was document was imported into NVivo version 11, a qualitative data analysis software program.^30^

The data were analyzed in accordance with the guidelines of Braun and Clarke (2006) for thematic analysis.^6^ Coding was conducted by assigning a code to each extract and named according to relevance of the concept and assembled into potential themes and subtheme categories. Codes were reviewed several times for familiarity. Points of interest and potential themes and subthemes were highlighted. Relationships between the themes and subthemes were reviewed against the data set. Themes and subthemes and their names were first reviewed independently by K.N. and subsequently peer reviewed and discussed by A.J. and E.W. This iterative process involved amending theme names and content as analyses developed to ensure that themes best fit interpretation of the data. The analytical approach was data driven, and authors did not have any pre-existing expectations regarding which themes would be generated from analyses. A constructionist framework was used to portray the meaning of the reality of the posters in the study. A constructionist framework argues that the “way in which the world is understood is related to specific social, cultural contexts” (*p*30).^5^ In other words, we were mindful that when analyzing the interactions, we would inevitably be viewing them through our own social and cultural context, not that of those posting on the forums.

## 3. Results

Two main themes were identified. The first theme, “The informal rules of exchanging and receiving network support” describes the communicational exchanges between posters on the forum and the informal rules that posters abide by when sharing experiences. The second theme, “Parents positioning themselves as experts” highlights how parents presented themselves, rather than health care professionals, as experts in their young person's pain.

### 3.1. The informal rules of exchanging and receiving network support

Parents posted to the forum to seek both information and support regarding their experience of parenting a young person with CRPS. For example, parents, such as Joanne (below), joined an existing CRPS discussion thread to seek help from parents who shared similar experiences, highlighting the importance of both seeking and providing support among the online parental community.

Can anybody help us please? My daughter fractured her ankle 5 months ago as a result of tripping on a pavement. She was in and out of plaster for 10 weeks whilst orthopedics decided whether it was fractured or not… Her pain is so bad now that she's back on the children's ward at hospital on intravenous morphine, gas & air. The staff have just had to sedate her to let her get some sleep. My daughter can't even start any physiotherapy as the pain is too great. What do we do now? Has any other child with CRPS been in this level of pain? We are desperate for any help and advice. (Joanne)

Focusing on online forum parental responses for assistance, posters were receptive and helpful to fellow parents in their responses providing multiple forms of support. Using the example of sharing details of a recommended book below, Jane and Anne demonstrate how posters sought out interaction with others displaying positivity, familiarity, and understanding of each other's shared experiences. Jane's message below demonstrates her desire to provide both informational support to a fellow parent of a young person with CRPS, but also empathy through her comment of “hope things get better soon”:

I've got an excellent book which has helped my knowledge. I'll get the name of it if you want. You can private message me later and I can provide more information about services that may be beneficial. Good luck. (Jane)

Thank you, Jane… The book title would be wonderful thanks so much. (Anne)

I so feel for you. It's such a nightmare feeling so helpless. The book is called (Name of book). I will private message you about the other information. Hope things get better soon. (Jane)

As the online friendships developed over time, a sense of responsibility emerged. This was demonstrated through parents providing apologetic explanations for absences from the forums, indicating a sense of needing to justify why they had not “been there” for their cyber friends. For example, an ongoing exchange had been taking place between Mary, Sarah, and Susan focusing on Mary's experiences of parenting her daughter with CRPS. Sarah and Susan showed both concern and emotional investment in Mary's experiences. Mary had been absent for the latter part of the exchange and offered an explanation for her temporary absence from the discussion. This could indicate Mary's sense of needing to conform to the social rules of the friendships she had made online, and in so doing, reciprocate the emotional support provided by fellow posters. Support was offered and elicited between multiple forum members as exemplified by the following 3-way interaction between Susan, Mary, and Sarah:

My understanding is the worse thing to do is immobilise so somehow there needs to be some movement going on. (Susan)

Definitely keep it moving the cast was a nightmare!!! (Sarah)

Apologies, I meant to reply yesterday but was stuck in Accident & Emergency the whole day. We're trying to get her (daughter with CRPS) to straighten her leg as much as we can but it causes intense pain, it's very distressing to watch. So, worrying! (Mary)

Mary's explanation of her absence and lack of communication is clear and exonerates her from any potential offence caused by her not acknowledging the support offered. She also shares information about the process her child was undergoing and expresses her distress at being unable to do anything other than “watch.” In so doing, Mary conveys the emotional and practical challenges associated with caring for a young person with CRPS and indirectly demonstrates her right to hold membership of this distinct online group.

Given the unpredictable nature of CRPS symptoms and response to treatment, it was difficult for parents to offer anything other than empathic or social support. This is exemplified by Susan below who expressed that

To be honest, I wasn't sure whether to reply to this message as I'm not sure if I could help a lot. My thoughts really go to your son. All I can say is if he can keep mobility whilst on crutches tell him he's doing amazingly well!! (Susan)

These data also exemplify parents managing the uncertainty of whether or not they feel they can help each other, which is strongly linked to the fluctuating nature of the condition. What is important about this is that even when parents had no practical suggestions, they found alternative ways to support one another. It was more important to respond to a parent in distress, with a form of support, even if this was not the type of (practical) support sought. Provided support often mixed empathetic and social with practical, exemplifying the value of all 3 types. Elizabeth stated

I have such sympathy for both of you. It is the hardest thing to not be able to help your child in pain or physically comfort them which causes them to scream aloud in pain. I really hope the information I provided above about drugs and treatment really do help you. (Elizabeth)

It is important to consider the credibility of sources of support, which were both requested, and offered between parents of youth with CRPS. In this study, evidence-based books on pain management authored by experts in the field were suggested by posters to fellow parents (see above post regarding the name of the book). However, non-evidence based information concerning analgesia and therapies for treating CRPS in youth (eg, lightning therapy) was also discussed online. Consequently, the evidence base for sources of information discussed online was mixed.

### 3.2. Parents positioning themselves as experts

The forums also provided parents with a space to explore their relationship with the medical profession. They were able to demonstrate the importance of the expert knowledge that they hold in relation to their child, which they did not always feel was recognized by the medical profession. On these occasions, parents presented themselves as “experts” compared with the health care professionals. They were then viewed by definition as “good” parents because they knew what their child needed and were prepared to fight health professionals to ensure that their young person's needs were met

After battling with the medical team (they seem to believe physiotherapy and active coping is the best way forward) we were given this medication which we have now increased by double. Son's constant chronic pain reduced from a 9/10 to a 6.5/10. He's managed a full night's sleep which is tremendous progress!! (Margaret)

Margaret justified her decision to “battle” the medical team by showing that her fight resulted in a reduction of their young person's pain. Indeed, sharing these experiences allows posters to demonstrate their perceived greater knowledge of CRPS, often highlighting this by strong negatively valenced adjectives to describe the medical profession. For example, Sarah portrayed herself as being superior to the *“*floundering*”* consultants. Use of the word floundering suggests Sarah's perception of the consultants as indecisive and to be experiencing difficulty, enabling her to position herself as the expert in terms of her son's care. Posters who adopted this position also displayed their perceived superiority by providing advice to other parents. For example, Susan adopted an authoritative role within the online conversation, clearly advising parents less experienced in managing their young person's CRPS about next steps in supporting their young person. Susan also felt the need to provide informational advice although the poster did not request for informational support. This further extends her role as an expert as she assumes that the other posters will want to know the knowledge and advice she has to offer even if they have not requested it

Getting the correct diagnosis is essential! It usually starts with an injury (Has there been one)? I would personally recommend a very good osteopath to examine if there's anything else causing it. (Susan)

Parents adopting this “expert” role often sought a detailed description of symptomology and causation, suggesting certain treatment regimens or an alteration of lifestyle factors. An example of this is provided by Catherine mimicking the role of the health care professional, assessing, diagnosing, and formulating a treatment plan

I'm wondering if your daughter experienced symptoms in the evening? I know it was a long time ago but was thinking if she could have had pins and needles or an infection of some sort, possible excess sweating on that night? It might be beneficial for her to take some vitamin C. However, there are differing reports on the effectiveness of it and the optimal times to take it allegedly can cause early onset of CRPS? (Catherine)

Parents adopting the “expert” role suggested means of identifying “good” and “bad” health care professionals. In so doing, they clearly expressed a perception of their knowledge about CRPS, their young person's CRPS journey, and treatment outcomes as being superior to that of medical professionals.

Don't let the physiotherapist push your son through the pain barrier. CRPS is different to any other chronic pain condition. Physiotherapists which are experienced in CRPS will exercise to the pain barrier limit and not beyond. (Sarah)

Sarah's post conveys a sense of dictation to other posters. The content of Sarah's post infers that parents who are inexperienced don't understand exactly which health care professionals are equipped to treat pediatric CRPS, and which are not. This of course has implications in terms of their parents' ability to support their young person's recovery from CRPS. Parents perceived to be “good” are knowledgeable, and in particular, are responsible for knowing the difference between experienced and inexperienced health care professionals specializing in CRPS. This in turn means that these “good” parents ultimately know best how to reduce their young person's pain over health care professional's advice.

## 4. Discussion

The aim of the current study was to qualitatively explore how parents of young people with CRPS communicated with one another using online forums. Two main themes were identified: “The informal rules of exchanging and receiving network support” highlighted the “unofficial” social conventional rules that posters abided by when communicating with fellow parents. The second theme, “Parents positioning themselves as experts” emphasized the power differentials amongst the posters on the online public forum threads.

Communication between parents was overwhelmingly supportive, with informational and empathic support being the most dominant support offered and solicited. For parents, it seemed that the act of self-disclosing information about their own personal story meant that they were then able to elicit support from fellow parents. Sharing information online enabled fellow posters to offer solutions drawn from their own experiences of managing similar challenges. Perhaps most interestingly, support was offered and elicited between multiple forum members on a single thread, highlighting a different communication dynamic to a face to face conversation between 2 or 3 individuals. These findings are similar to previous online forum studies assessing parental experiences of caring for a child with a long-term health condition (cancer and neuromuscular disorder), which found that elevated levels of self-disclosure in posters on the forum resulted in high levels of emotional support being offered amongst posters. Posters also established their legitimacy to be part of the support forum and post messages by sharing their personal experiences.^11,27^

Study findings highlighted that posters perceived the need to self-disclose, to provide a high level of information in their posts and to adhere to social etiquette rules to feel accepted by fellow forum posters. Such findings are consistent with those of previous online forum studies with adults with rheumatoid arthritis^1^ and CRPS,^40^ where posters shared personal experiences to establish their role on the forum and to be accepted and valued as a fellow forum member.

One of the most difficult things for parents in this study concerned having to sit with uncertainty associated with their young person's CRPS. This was exemplified by Mary when discussing her experience of sitting in Accident and Emergency (Emergency Room) with her daughter being unable to do anything other than watch, feeling uncertain about her daughter's future and prognosis. Such uncertainty was experienced in other ways such as feeling confused about where to seek reputable information and support, or a perceived sense of their young person's symptoms not being believed by others. Such an experience fits with the wider adult chronic pain diagnostic uncertainty literature^35,36^ and studies, which have articulated parental frustrations around credibility of their young person's pain and with a constant search for a diagnosis.^25,29^ We identified consequences of this uncertainty, which differed among parents. Some parents felt that the only support strategies they could offer related to emotional support and sympathy as informational strategies were unavailable. By contrast, other parents became empowered in the face of uncertainty, perceiving themselves to be experts with regards to their extensive knowledge and understanding of CRPS. These parents challenged the biomedical model of authority,^3^ which typically questions parentally reported opinions. Interestingly, this second group in effect reversed the model and explicitly challenged the advice from health care professionals, provided in response to young people with CRPS and their parents. The uncertainty around diagnosis itself seemed to fuel the parents' confidence to challenge the medical opinions and take control over the care of their young person. These “expert” posters have defined themselves as knowledgeable and resourceful in treating their young person's CRPS. This seems to go beyond caregivers feeling tension between their own knowledge being centralized while still wanting the authority of biomedically given knowledge. However, the comments about “floundering” consultants could suggest that they would prefer to feel that medics do offer authoritative, useful knowledge. We need to know more about enabling parents to feel that their patient-centered narratives can be fruitfully combined with biomedical accounts and whether online forums could be useful places to reflect such fruition.

Although forums in this study were not designed to fulfil a CRPS support group function, communication between posters identified a clear sense of social conformity and established power dynamic with regard to the social nature of the online interactions. Specifically, certain threads were dominated by particular posters, evidenced by their sense of asserting power and offering advice to fellow posters, even if it had not been elicited on some occasions. These posters' positions of power can be likened to the theory of a community of practice^45^ where a community have the same common concern and set of problems and through interaction on an ongoing basis, expertise and the knowledge in this area is expanded. In this study, the knowledge of forum posters comprised the “community of practice,” with all members sharing the same challenge of parenting a young person with CRPS. Interaction of forum members online to share experiences and solutions to very specific challenges associated with managing their young person's CRPS condition enabled the group members to develop and expand their knowledge and expertise concerning parenting in the context of having a young person with CRPS.

This study includes some notable strengths. First, the study provides important information regarding both the nature and function of naturalistic communications between parents of young people with CRPS over time on online forums. This focus on communication style is particularly novel and important in an increasingly digital age. Second, it provides novel information that can enable improved understanding of the support needs of parents of young people with CRPS. However, the study is not without limitations. One particular challenge concerns that of demographic data of posters. The anonymous online nature of the forum data means that we do not possess demographic data for participants, eg, age, gender, and ethnicity. A second limitation concerns the inclusion of 2 UK forums in this study, comprising a total of 107 posts from 39 posters. Future research could helpfully include a larger number of forums in multiple countries to further explore parental experience.

With regard to the study implications, our findings suggest the need to provide better emotional support for parents to enable them to manage their own needs and also those of their young person. Our analyses suggest that it is possible that this need identifies an opportunity for health care professionals to become more involved in supporting parents by promoting and directing parents to reputable sources of support. Furthermore, the study has suggested how parents can indeed provide peer support to fellow parents of young people with CRPS. Peer mentorship programs have been shown to be effective in terms of pediatric pain-related conditions.^37^ Although we appreciate that these findings are extrapolated from research conducted with young people rather than adults, it is possible that a peer mentorship model may offer some ways in which parental support can be formalized in online spaces to better support parents of young people with CRPS.^45^ Future research could helpfully consider how we might encourage good peer mentorship practice in supporting parents of young people with CRPS.

In conclusion, study findings have demonstrated that individuals parenting a young person with CRPS perceive online forums to be useful spaces for sharing their experiences and seeking support from peers. Sharing personal stories and challenges enables parents to normalize and better understand their experiences with parenting a young person with CRPS and also gain support from parents who have shared experiences and knowledge. By identifying the way in which parents seek online and why and how they use this information, this can be used to address the information missing in the health care and treatment plans for young people and also address the emotional, physical informational needs of parents directly.

## Disclosures

The authors have no conflict of interest to declare.
